# Supplementary figures and images for: A method for analysing tissue motion and deformation during mammalian organogenesis
Source: PLoS Comput Biol. 2025 Oct 15;21(10):e1013275. doi: 10.1371/journal.pcbi.1013275 (PMC12548895; doi:10.1371/journal.pcbi.1013275)

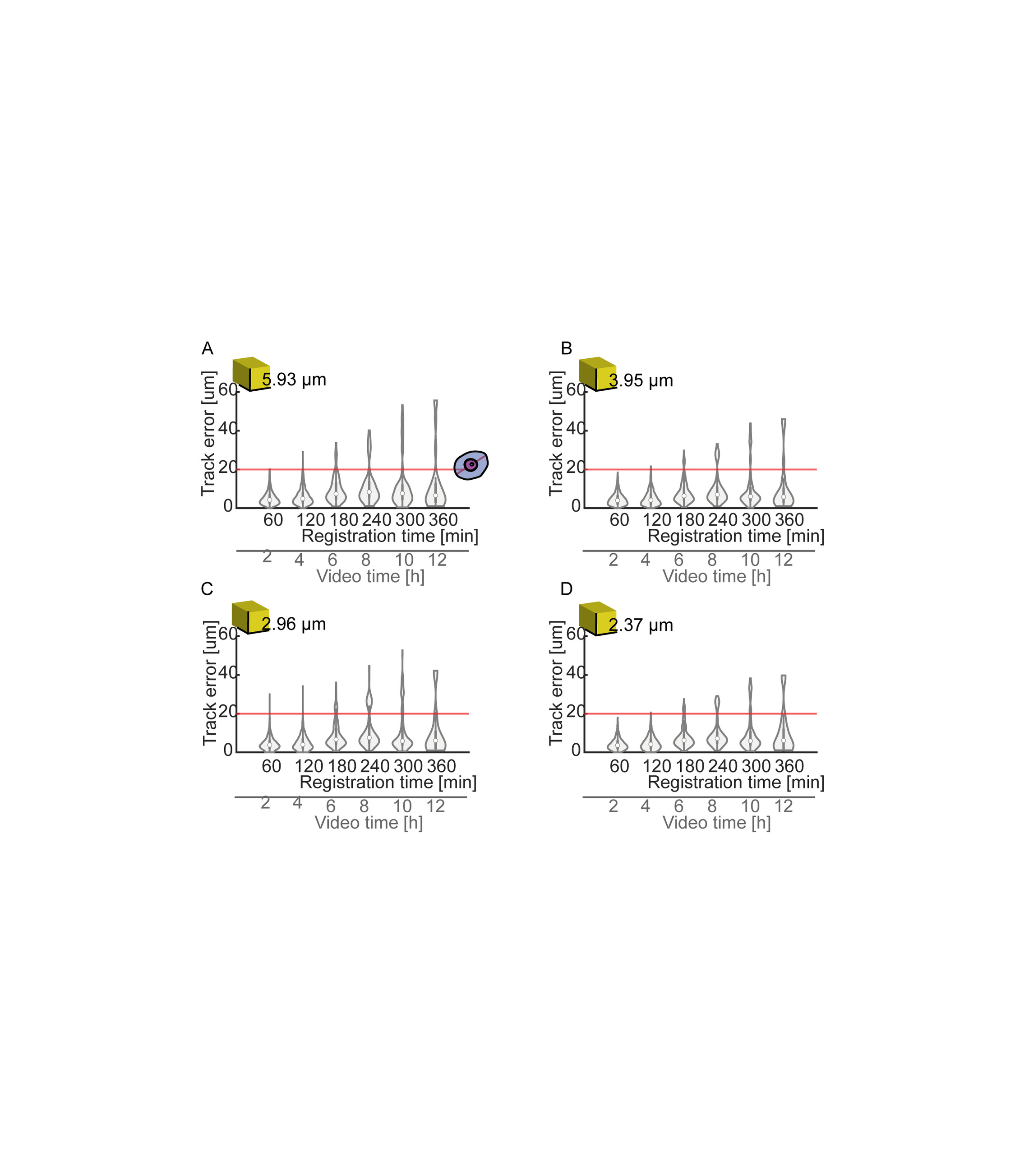

Supplement: S1 Fig — The sequential error was estimated for the ground-truth points when the image was resized at 10%(A), 15%(B), 20%(C), and 25%(D). The error is expressed in μm. The red line indicates the averaged cell diameter of 20 μm. The yellow cubes indicate the voxel size. The registration time is half of the video time, according to the registration strategy adopted. The graphs represent the errors of 9584 tracked cells in the images of 9 embryos (e01, e02, e05, e06, e15, e16, e24, e26, e27). (TIF) [file pcbi.1013275.s001.tif]

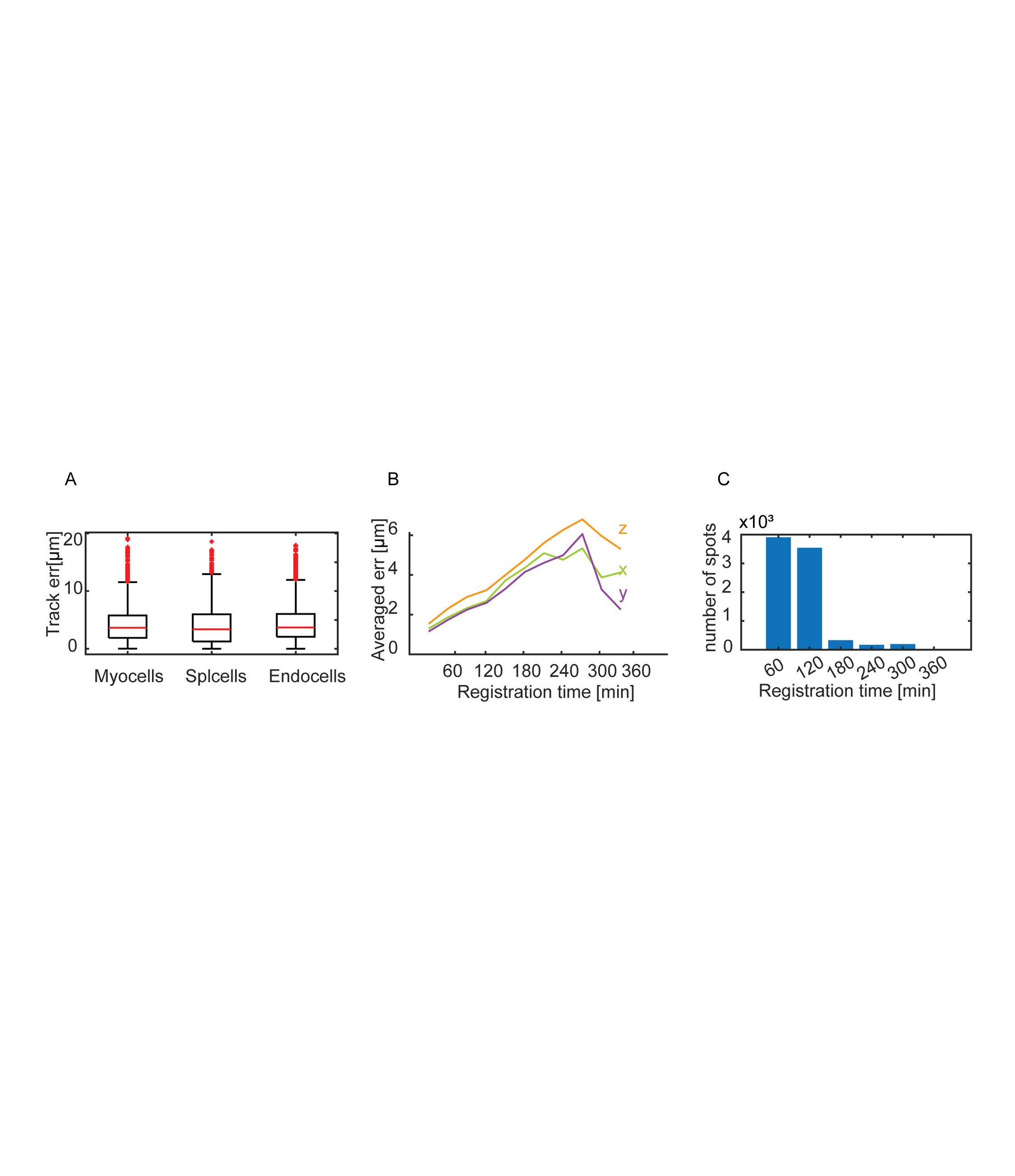

Supplement: S2 Fig — (A), The error made in continuous registration of landmarks belonging to different cardiac layers from images at 25% resolution. There is no statistically significant difference between the error made on myocardial cells, splanchnic mesodermal cells, and endodermal cells. (B), x-y-z decomposition of the continuous mean error, related to a 25% rescaled image (e01, e02, e05, e06, e15, e16, e24, e26, e27) is reported. (C) Number of landmarks in two-hours video interval for which the error was estimated. (TIF) [file pcbi.1013275.s002.tif]

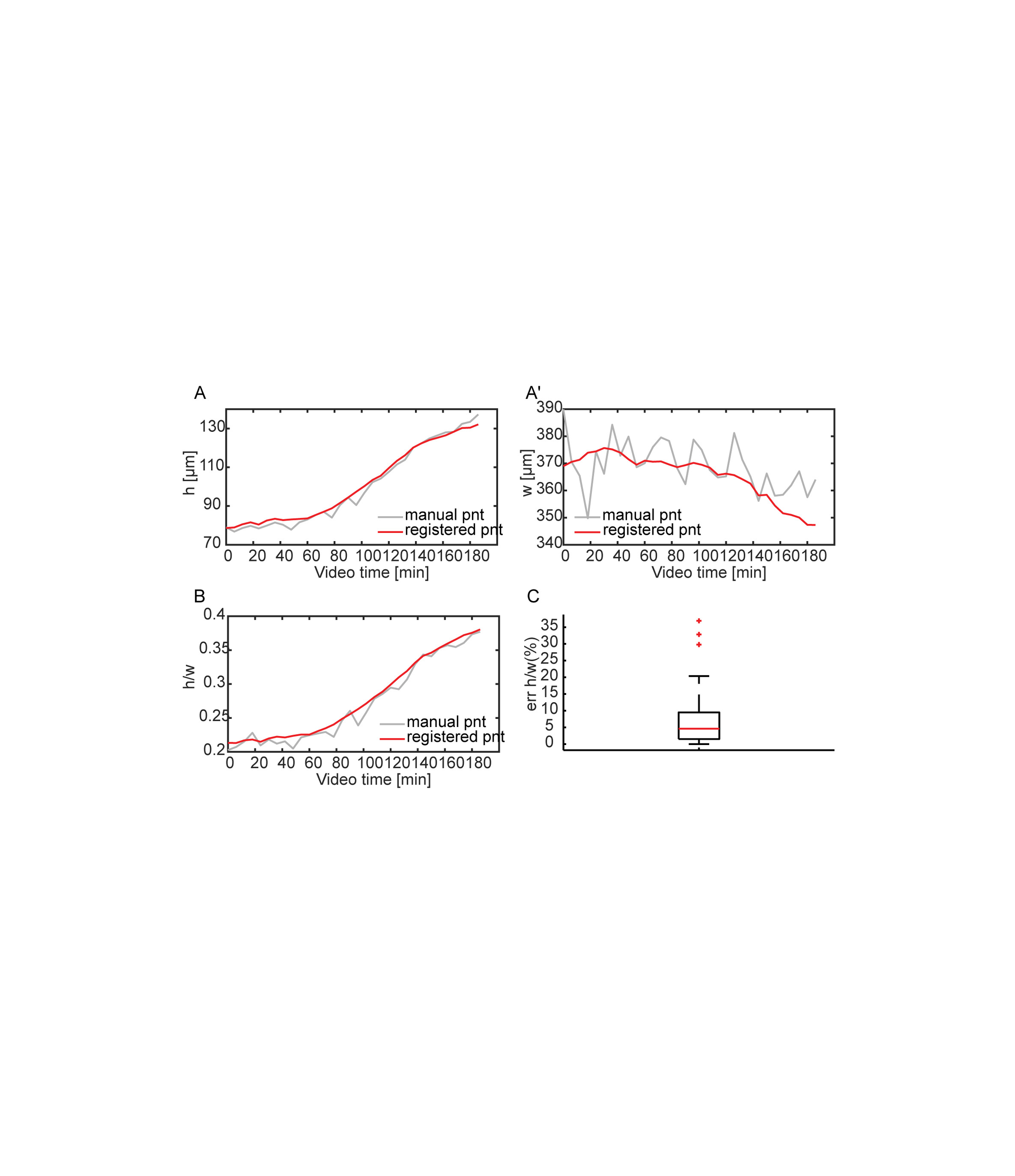

Supplement: S3 Fig — The data are related to a single embryo (e01). (A, A’), In red the h and w lengths between landmarks taken manually on the shapes at each frame. In grey, the lengths h and w calculated automatically for each frame at the selected landmark at time t0 (registered point). (B), Comparison between the feature h/w obtained from the manual landmarks (red line) and the landmarks defined by the continuous model (gray line). (C), Percentage difference between the two features performed on 3 embryos (e01, e07, e27). (TIF) [file pcbi.1013275.s003.tif]

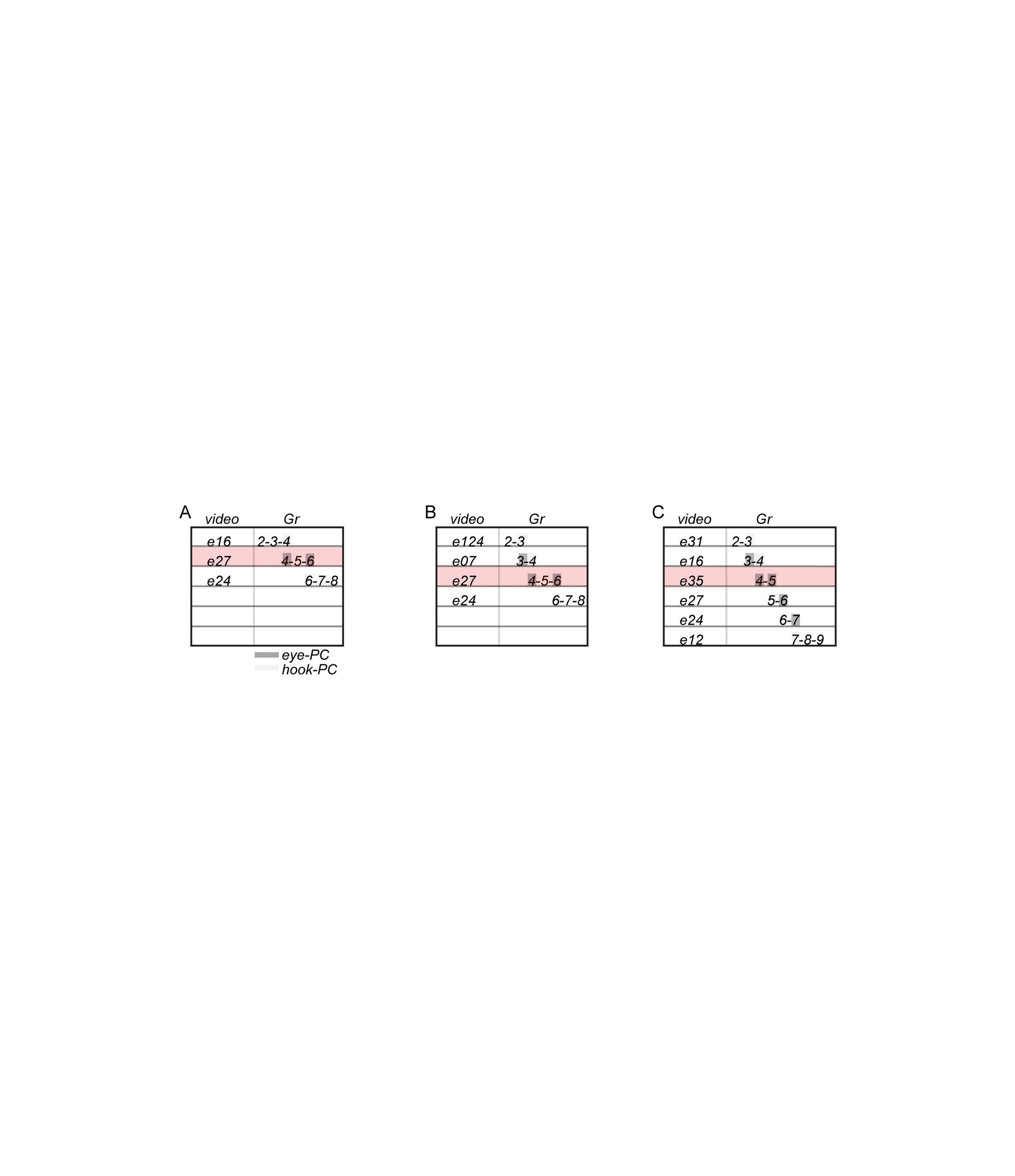

Supplement: S4 Fig — We evaluated the growth profile for the 10 zones between Gr2 to Gr8 for three different concatenation paths, shown in (A),(B),(C). We compare Gr2 to Gr8 as it represents the common stage range among the three different paths. The matching score between the growth profiles of the model and the one extracted from the live image was 62%, 65% and 81%, respectively. We chose the last concatenation path because it showed the highest match. (TIF) [file pcbi.1013275.s004.tif]

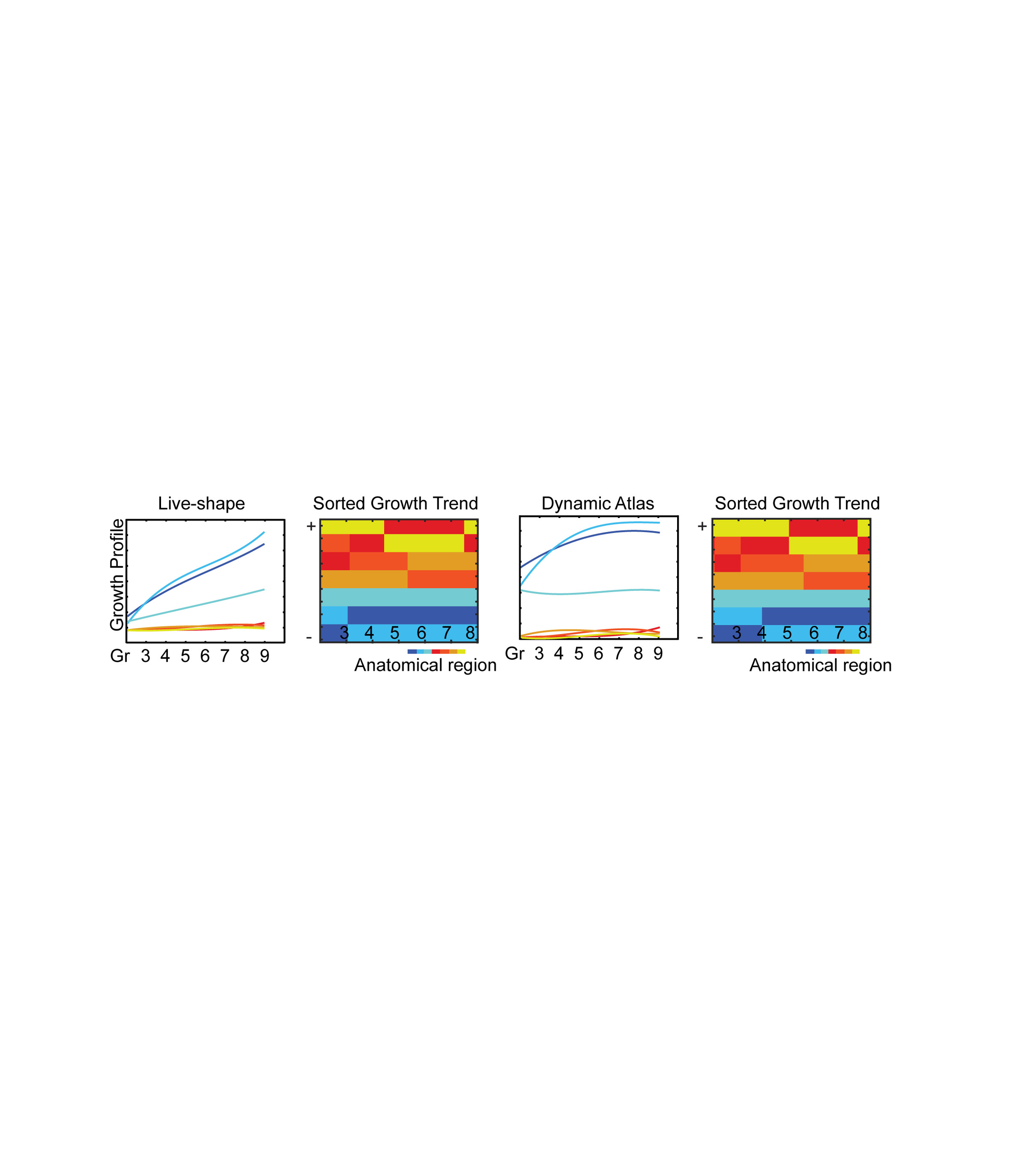

Supplement: S5 Fig — The growth profile for the anatomical regions (excluding regions 1,5, and 10) were computed for the live-shapes and the Dynamic ATlas, with the corresponding heatmap. The two sorted growth trend matches at 92%. (TIF) [file pcbi.1013275.s005.tif]

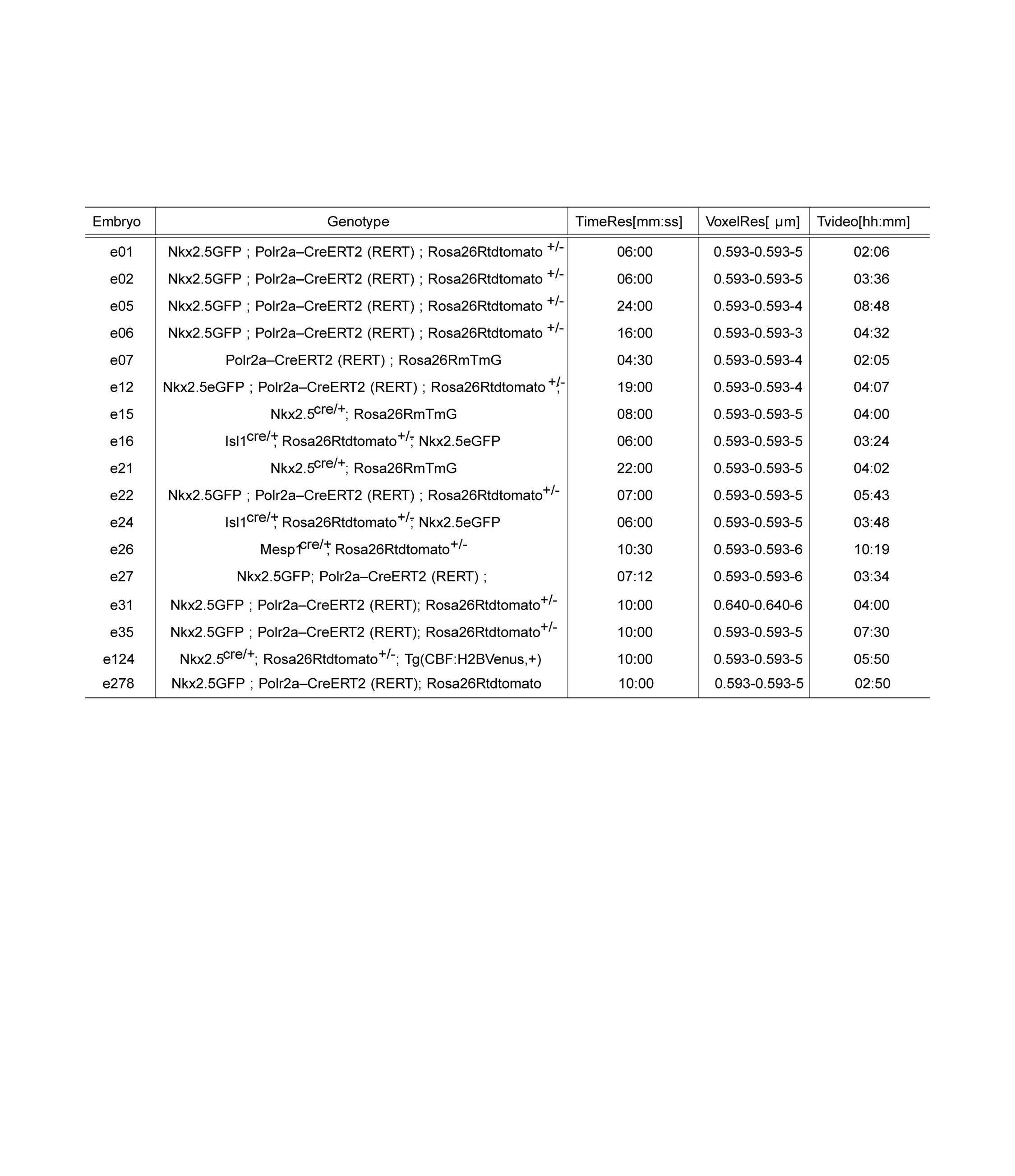

Supplement: S1 Table — The first column shows the list of embryos involved in our study; Genotype column reports the mouse allels (Nkx2.5GFP [[34], Polr2a–CreERT2 (RERT) [35], Nkx2.5cre/+ [11], Rosa26Rtdtomato + /- [36], Rosa26RmTmG [37], Tg(CBF:H2BVenus,+) [38]); TimeRes column lists the time resolution of the time-lapse[mm:ss]; VoxelRes column indicates the resolution in μm along the x,y,z axes; the last column (Tvideo) shows the total duration of the time-lapse[hh:mm]. e278 in grastrulation phase. (TIF) [file pcbi.1013275.s006.tif]

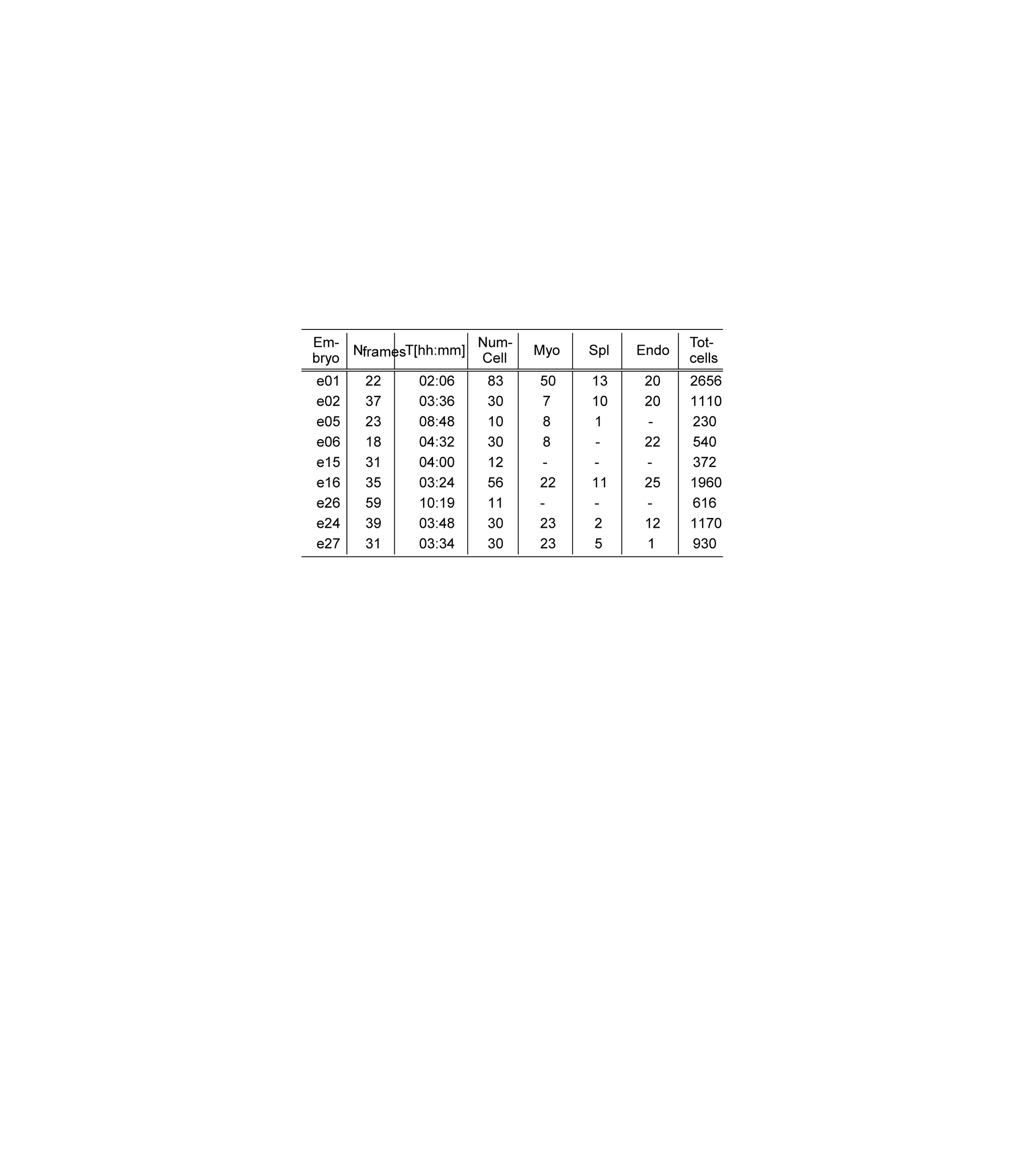

Supplement: S2 Table — We have indicated the number of frames for each video (Nframes), the duration of the video (T[hh:mm]), the number of totals tracked cells (NumCell). We have indicated which of these cells belong to the myocardium (Myo), how many to the mesoderm splachnic (Spl) and how many to the endoderm (Endo). In Totcells the total number of points, i.e., cells, for which the error is quantified. The total is given by the number of cells Numcells*Nframes. For e05 and e15, 1 and 4 endothelial cells were tracked respectively. (TIF) [file pcbi.1013275.s007.tif]

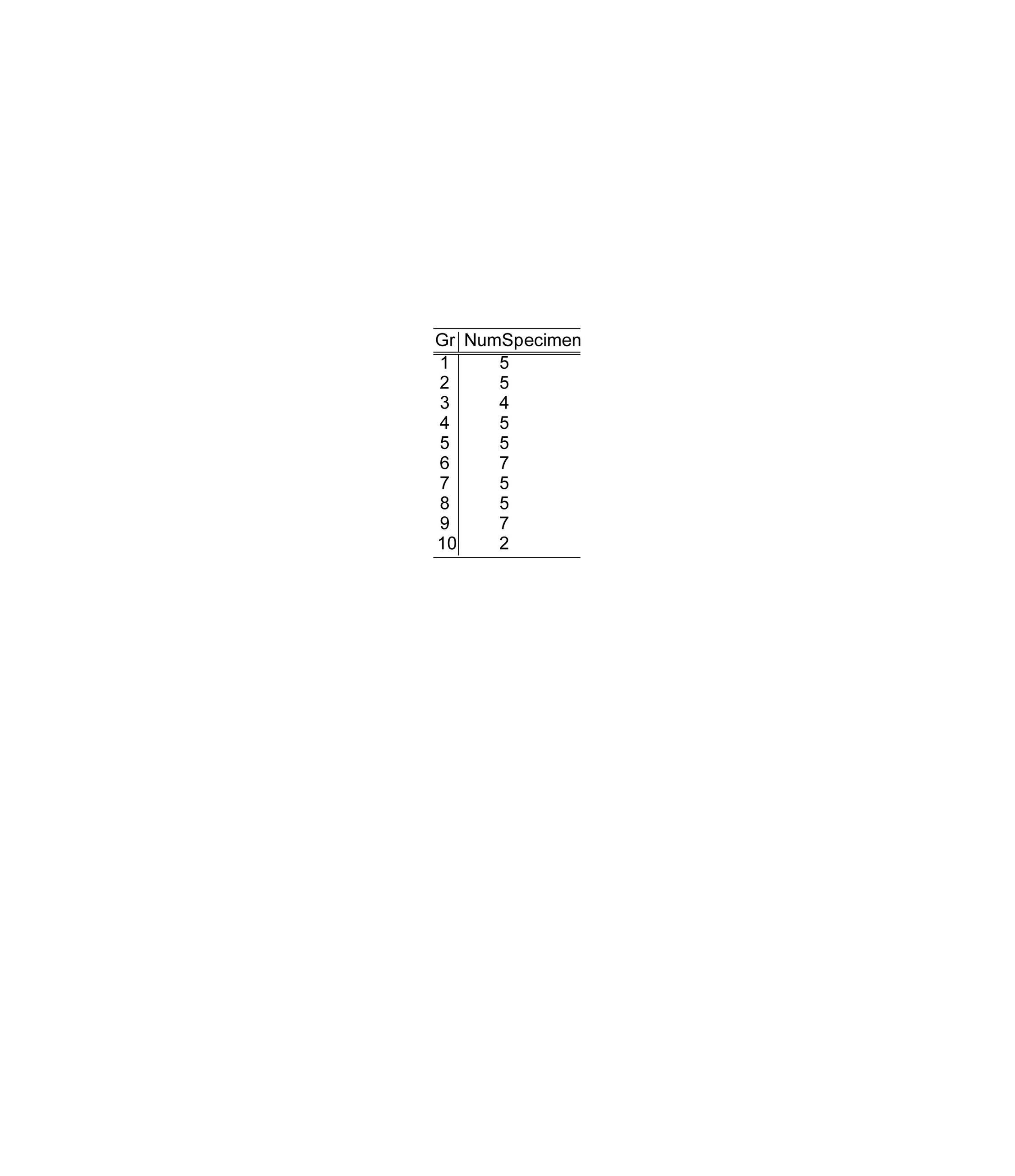

Supplement: S3 Table — Number of specimens used to build the Atlas Grs [9]. (TIF) [file pcbi.1013275.s008.tif]

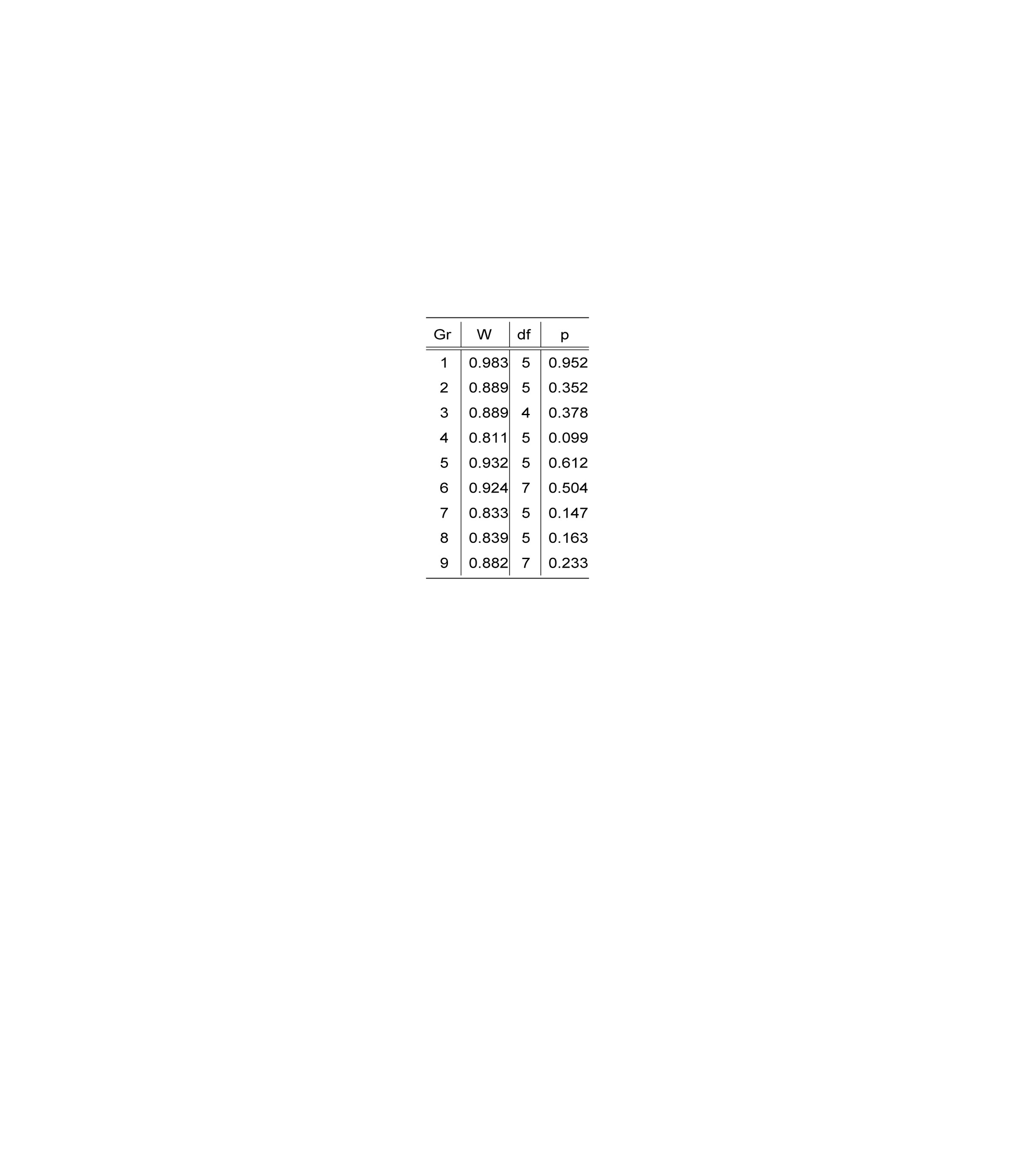

Supplement: S4 Table — (TIF) [file pcbi.1013275.s009.tif]

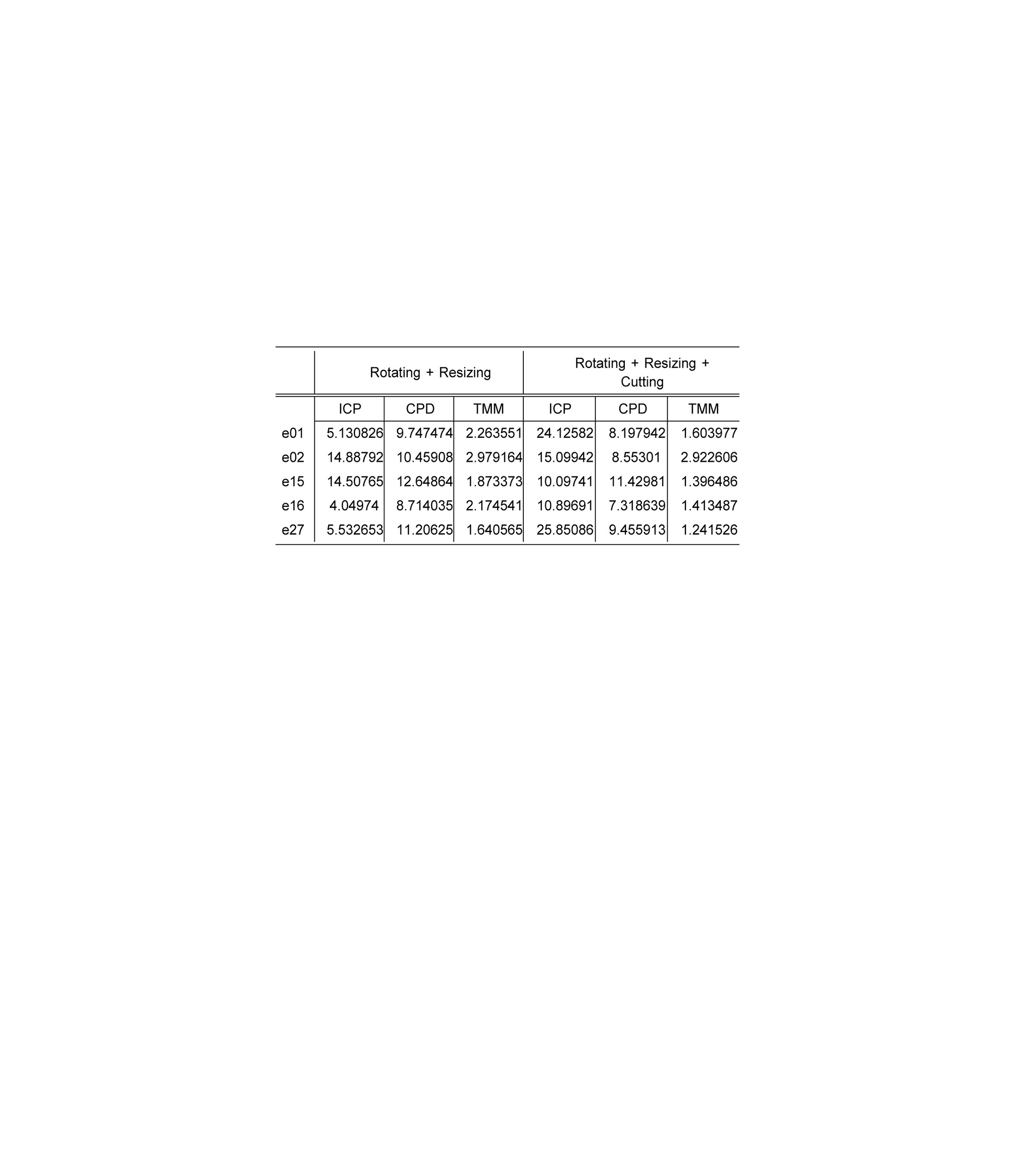

Supplement: S5 Table — In each column, the rmse is reported for each embryo at each registration technique (ICP, CPD, TMM). We assessed the performance of the most popular methods for rigid point set registration: ICP [39] and the CPD [40] and TMM-based [20] algorithms. To this end, we evaluated how well the algorithms aligned a shape to a modified version of that shape. The altered shape underwent three modifications: 1) a random reduction of 30% of its points. 2) a rigid transformation as a rotation of 20° around zaxis; rotation of 70° around the x-axis; a rescale of 20% with respect to the original shape size. 3) a partial cut of the IFTs. The algorithms were tested to the first frame (t0) of five different embryos (e01, e02, e15, e16, e27) by calculating the root-mean-square error (rmse) between the original shape and the corresponding points in the altered one We found that the TMM-based algorithm performed better compared to ICP and CPD algorithms. So, we decided to implement the TMM-based algorithm as the first step in the spatial mapping pipeline. (TIF) [file pcbi.1013275.s010.tif]

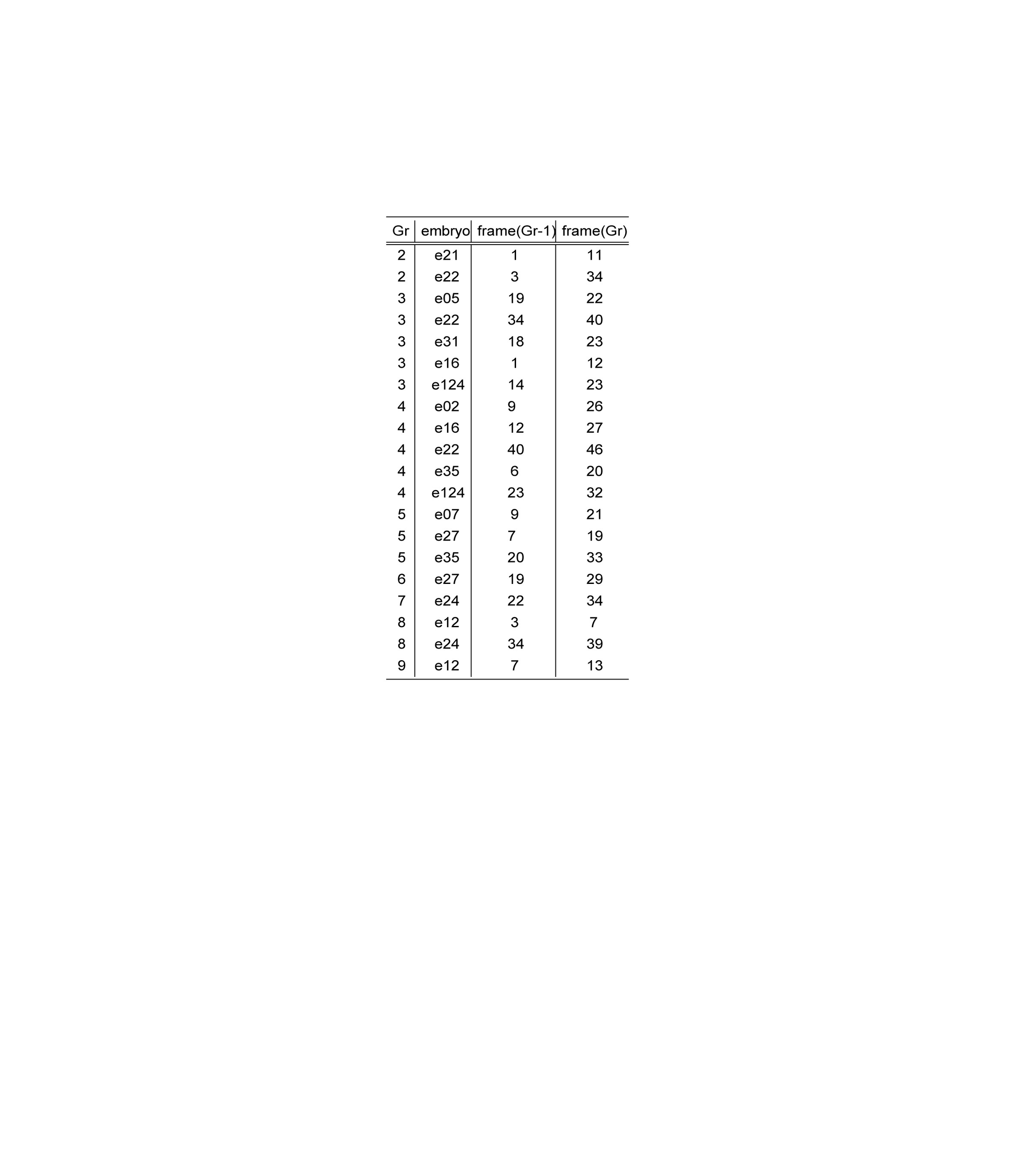

Supplement: S6 Table — The first column shows the Atlas group(Gr) associated with the live embryo shown in the second column. The last two columns show the video frames related to the rest state (frame(Gr-1)) and the deformed state (frame(Gr)), respectively. (TIF) [file pcbi.1013275.s011.tif]

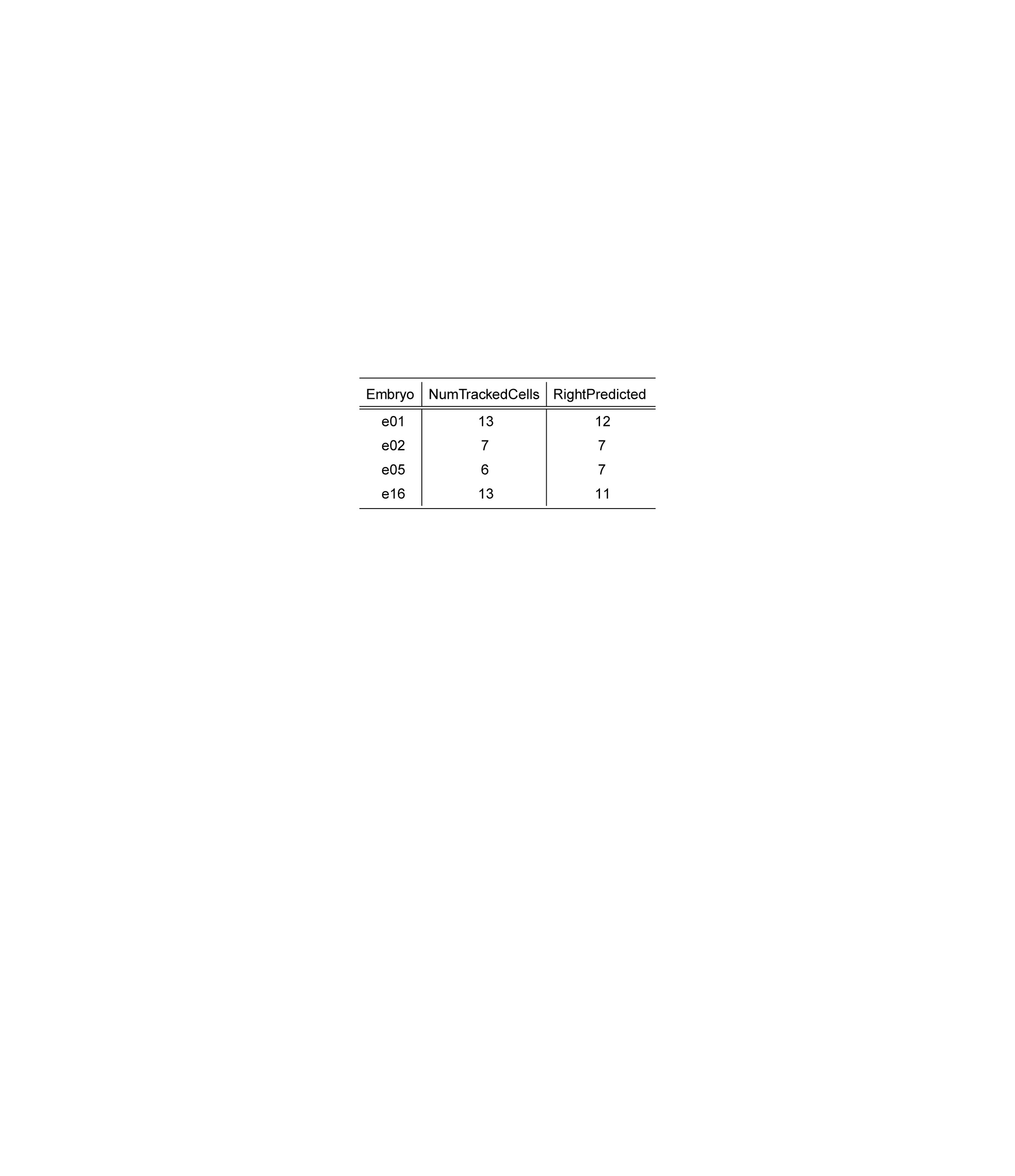

Supplement: S7 Table — (TIF) [file pcbi.1013275.s012.tif]
