# Supplementary material for: A method for analysing tissue motion and deformation during mammalian organogenesis
Source: PLoS Comput Biol. 2025 Oct 15;21(10):e1013275. doi: 10.1371/journal.pcbi.1013275 (PMC12548895; doi:10.1371/journal.pcbi.1013275)
Supplement: Attachment — Submitted filename: Response to Reviewers.docx [file pcbi.1013275.s016.docx]

Responses to Reviewer #1

We thank Reviewer #1 for their positive assessment and for the critical questions that have helped us to significantly strengthen our manuscript.

*Comment 1: Authors describe an atlas-based alignment using non-rigid registration. Please discuss how this affects to the deformation maps computation as the non-rigid registration can compensate inter-specimen variability of deformation patterns, growth and movements (including intercalation).*

We thank the reviewer for this important observation.

We fully agree with the reviewer, applying non-rigid registration before analysis would indeed artificially "flatten" inter-specimen variability in deformation, growth, and movements that we aim to quantify. Our entire workflow was specifically designed to avoid this critical artifact.

Our revised text now explicitly details the pipeline's sequence:

1. Dynamics are calculated first on native geometries. For each embryo, we compute the stepwise motion vectors and deformation maps (growth, anisotropy) directly from its original, staged 'Live-Shape' sequence. This step captures the authentic, unaltered dynamics of each specific specimen.
2. Non-rigid registration is used last, solely for spatial mapping and averaging of the parameters extracted from each specimen. The registration's purpose is to establish anatomical correspondence between each point on an embryo's 'Live-Shape' and a corresponding point on the common Atlas reference. This process does not alter the pre-computed deformation or motion values.

Therefore, the Atlas acts as a 'common canvas' for visualizing pre-computed, individual data. The non-rigid registration standardizes 'where' the data is displayed (the spatial context for comparison) but does not alter 'what' was measured (the deformation) or 'how' it moved. The stepwise trajectories visualized on the Atlas are the authentic trajectories from the 'Live-Shapes', simply re-plotted and averaged in a common reference frame. The cumulative deformation, in turn, is the product of these individual deformation steps, concatenated within the Atlas framework.

Regarding cell intercalation, our continuum-based method measures net tissue deformation and does not explicitly resolve individual cell rearrangements. However, the high accuracy of our method in the heart, contrasted with its poor performance in highly fluid tissues like the gastrulating mesoderm (Fig. 2M-M''), provides strong indirect evidence that large-scale, disordered cell intercalation is not the dominant mechanism driving heart tube formation. This is further supported by previous studies of cell clonal analysis showing the coherence of clonal groups in the developing myocardium.

Finally, while validating the anatomical accuracy of the non-rigid mapping itself is intrinsically challenging, we implemented a qualitative validation protocol. As clarified in the text, this involved an expert review that used local mesh area as a proxy to confirm that anatomical features were consistently mapped (Fig. 3F).

We have substantially revised both the subsections “Registering Individual Shape into the Atlas Reference Geometry” (lines 419-422, 441-447) and “Extracting Stepwise Tissue Deformation sections” (lines 472-507) to fully clarify the discussion above.

*Comment 2: It is not clear if the motion estimation and deformation estimation are done before the template /atlas registration or after. Please clarify and discuss.*

We thank the reviewer for pointing out this crucial point.

Our entire pipeline is built upon a strict, sequential process, which we have now made explicit in the manuscript:

1. Motion Estimation is performed FIRST, on native geometries. As now detailed in the section 'Estimating Individual Live Image Motion', the dense motion field ({Ti}) is estimated directly from each raw live video for each embryo individually. This initial step captures the authentic dynamics and yields a 'Live-Shape' sequence for each specimen, entirely independent of the Atlas.
2. Atlas Registration is performed NEXT, for synchronization. The individual 'Live-Shape' sequences are then synchronized in time (staging) and space (mapping) using the Atlas as a common reference. The sole purpose of this step is comparative: to assign developmental stages to frames and to establish a common coordinate system for visualization.
3. Deformation is calculated LAST, using the original shapes (‘Live-Shape’). We have completely rewritten the section 'Extracting Stepwise Tissue Deformation' (lines 472-507) to clarify this critical point. The deformation is calculated from the original, native 'Live-Shapes' that are temporally aligned via Atlas staging. This calculation occurs before any spatial projection onto the Atlas. This sequence is essential to our method: it ensures that we measure the authentic biological deformation of each specimen, completely avoiding the registration artifacts that would otherwise be introduced.

Our method first quantifies the dynamics on the original data and only in a next step exploits the Atlas as a "common canvas" to compare and visualize these pre-calculated, unaltered results.

In the current version of the manuscript, we clarify the whole operational sequence of the pipeline. We have substantially revised the manuscript to better detail the steps described above.

*Comment 3: The optimization strategy does not look necessary as tracking methods already have tracking errors with maximal resolution. Please justify the need of reducing the resolution of the images. Is this a real-time application?*

We fully agree with the reviewer's observation. Reducing the image resolution is not a methodological need for our tracking algorithm, which indeed performs well at maximal resolution.

The resolution reduction is not a methodological requirement, but a pragmatic optimization aimed at balancing computational cost with tracking accuracy. While this is not a real-time application, the computational demands of processing our entire dataset, comprising long-duration 4D videos with full-resolution isotropic images often exceeding several gigabytes, are substantial.

Our goal was therefore to identify the lowest resolution that maintained an acceptable, sub-cellular tracking error. The analysis presented in Figures 2F and 2F’ (and detailed in Fig. S1) demonstrates that operating at 25% of the original resolution achieves this balance effectively. This approach significantly reduces processing time and memory requirements while introducing only a minimal impact on registration accuracy, with the error remaining well below a single cell diameter. This exploration of the minimal computational requirements for the obtention of accurate results will help the implementation by other laboratories, including those that may have limited computational capacities.

We have now clarified this rationale in the 'Optimization Strategy' section (lines 250-275). We explicitly state that this step is an optional but highly beneficial optimization for computational efficiency, and not a prerequisite for the method's accuracy.

*Comment 4: Authors should describe the interpretation of the two mechanical descriptors better: tissue growth rate (J) and tissue anisotropy (θ). It would be necessary to clearly define anisotropy of tissue dynamics. J and θ (as an angle) were already presented by Pastor-Escuredo et al iScience 2025 that presents deformation rates and cumulative deformation descriptors and ML analysis for fatemapping. A further description and interpretation and scope of the descriptors is required. Also the projection/statistical matching of the descriptors into the model should be better described.*

We thank the reviewer for this suggestion.

We have substantially revised the 'Extracting Stepwise Tissue Deformation' section to better contextualize the following points:

1. Biological Interpretation of Descriptors (lines 485-495). We have expanded the text to provide a precise physical and biological interpretation in the context of heart morphogenesis.

We have now clarified the physical meaning of our descriptors, which are derived from the principles of continuum mechanics as detailed in the Methods section (Principles of Finite Deformation Continuum Mechanics). We formally define J as the local rate of tissue area change (representing the relative change in area of each mesh triangle) and θ as the principal direction of maximal tissue stretch, which quantifies the anisotropy of the deformation.

We clarify that our continuum-based approach measures the net outcome of underlying cellular behaviours. Therefore, these descriptors represent tissue-level properties, not the specific cellular mechanisms (e.g., oriented division, cell shape change, cell size change…) that might generate them. This distinction clarifies the scope and power of our tissue-level analysis.

2. Clarified the Statistical Projection (lines 496-507): in this section we now better describe how these per-triangle, per-embryo descriptor values are projected and averaged onto the Atlas. We explicitly state that the face-to-face matching is used to transfer the J and θ values from each 'Live-Shape' triangle (facet) to its corresponding anatomical location on the Atlas. This process creates a standardized map of these descriptors for each specimen, which is the necessary step before computing the mean (µdef) and standard deviation (σdef) to reveal the consensus patterns of tissue mechanics.

*Comment 5: There is a lot of motion estimation description in the Results which can be valid, but there is little info on the statistical model. The statistical model should be further described as this is the most novel contribution of the methodology.*

We thank the reviewer for this important observation.

We should first clarify that our framework for tissue motion estimation is a deterministic descriptive model for spatiotemporal data fusion. The innovation lies in using the Atlas as a common coordinate system to deterministically concatenate individual, experimentally-derived motion profiles. So, the framework should not be considered as a statistical model – which would allow to make probabilistic inferences or predictions.

We have now substantially revised the text to clarify this crucial distinction. We explain that (lines 75-92):

1. The Atlas functions as a deterministic reference, a common coordinate system, that allows us to integrate the individual motion profiles from fragmented datasets. This integration is what enables the subsequent assessment of a consensus behaviour and its variability.
2. The final in-silico fate map is the direct result of this deterministic integration. It is a concatenation of the individual deformation steps in the common reference frame, providing a high-fidelity description of morphogenesis. We propose that creating this foundational, descriptive model is the essential prerequisite upon which any meaningful future statistical analysis or predictive models can be built.
3. The only truly 'statistical' components of our work are the descriptive statistics (e.g., the mean and standard deviation of the deformation). We compute these as outputs of our framework to quantify the consensus behaviour and its variability.

Furthermore, we explicitly state that a descriptive approach is the most scientifically rigorous choice for our dataset. Given the limited number of specimens per Atlas stage (on average 2-4, see Table S6), attempting to build a robust inferential or predictive statistical model would not be methodologically correct and it would lead to unreliable estimates.

To address this point and frame the future potential of our work, we have also added a new paragraph outlining this as a future direction (lines 728-742). This new section explains how, with a larger dataset, our framework could serve as the foundation for statistical models the reviewer was referring to.

*Comment 6: Authors should discuss better the limitations of doing cumulative rates onto a template/atlas instead of using trajectories (Lagrangian) as it has been done in other works. A better explanation of the dynamic atlas should be provided, it is a bit difficult to follow.*

We sincerely thank the reviewer for raising this important point, i.e. the core rationale of our work. We fully agree that a more explicit discussion of the trade-offs between our Atlas-based framework and Lagrangian approaches was needed. We have now fully revised the Discussion section (lines 659–666) to make this clearer.

1. Our motivation for an Atlas-based framework was strongly shaped by the practical challenges of imaging mammalian embryos. Long-term, drift-free recordings are extremely demanding, and many valuable datasets are incomplete, cut short by sample movement or with limited fields of view. Our approach was specifically designed to recover and integrate this imperfect but realistic datasets, which reflect what most developmental biology labs can routinely generate.
2. Mammalian development is highly variable across embryos at the spatiotemporal resolution of interest. Even with cutting-edge imaging, obtaining a large cohort of uninterrupted, long-term recordings is nearly impossible. A single “hero” embryo cannot capture the consensus behaviour, nor the natural range of variation. The Atlas addresses this by combining multiple shorter or partial recordings into a common reference, making it possible to build a population-level model.
3. Inter-embryo comparison requires a shared coordinate system. Early cardiac primordia lack stable intrinsic landmarks, so a Lagrangian analysis, although powerful, remains tied to each individual tissue shape, which we know is highly variable. The Atlas provides the essential external reference frame needed to align, average, and compare deformation patterns across specimens in a consistent anatomical context.

We would like to note that our strategy does make use of trajectories. We track the motion of randomly sampled points on the tissue surface, which closely follow cellular displacements. Deformation is not defined from cell-to-cell distances but from the evolution of each triangular element of the surface mesh (three neighboring nodes) as they move over time. Cumulative deformation is then obtained by integrating these stepwise changes, consistently formulated within continuum solid mechanics.

Our Atlas-based framework is not intended to replace Lagrangian analysis but to complement it in the data regime most common in mammalian developmental biology. It provides a practical and versatile toolkit to extract robust quantitative insights from fragmented, variable, and heterogeneous datasets.

We have also revised the 'In-silico Fate Map' section (lines 566-584) to better explain the Dynamic Atlas and its role as a tool for visualization and hypothesis generation.

The Dynamic Atlas is our solution to creating a single, continuous visualization that is conceptually similar to a full Lagrangian trajectory but constructed from fragmented data. While our cumulative deformation analysis averages motion profiles to find a statistical consensus, the Dynamic Atlas does the opposite. To ensure anatomical coherence and a smooth, physically plausible animation, it is built by concatenating the single, most complete 'Live-Shape' motion profile at each developmental transition.

Therefore, the Dynamic Atlas should be understood as a high-fidelity, descriptive composite, not a statistical average. It represents a "most-likely-path" reconstruction of morphogenesis, stitched together from the highest-quality segments of our experimental data. It serves as a powerful tool to generate hypotheses about cell trajectories, demonstrating how our framework can overcome data fragmentation to produce a seamless, kinetic representation of development.

*Comment 7: The discussion of the biological implications of the kinematic descriptors should be extended.*

We thank the reviewer for this important comment. A detailed discussion of the biological implications is indeed the primary goal of this quantitative analysis.

Due to the extensive methodological detail required to present our novel framework and, in parallel, the equally extensive biological validation and analysis it enabled, we made the strategic decision to present this work in two complementary manuscripts.

The companion manuscript, included with the submission to Plos Computational Biology and currently under review at eLife, is dedicated entirely to the biological implications derived from the methods we present here.

Specifically, using the kinematic descriptors developed in this paper, we reveal:

1. A strong spatial compartmentalization of tissue mechanics, where regions of high growth (predominantly in the lateral and ventrocaudal ventricle) are mutually exclusive from regions of high anisotropic stretch (in the medial stripe, oriented rostro-caudally).
2. A new model for heart tube morphogenesis, driven by the medial convergence of growing lateral tissue, which is constrained by two "belts" at the future OFT and IFT, causing the ventricle to bulge.
3. Evidence for the mechanisms driving cardiac looping, including a left-right bias in growth rates and potential compressive forces that could activate pathways like Hippo signalling.

To bridge these two manuscripts and address the reviewer's comment, we have now added a new paragraph to the Discussion (lines 708-715). This new text summarizes these key biological findings, providing the reader with a clear perspective on the utility of our method. It also explicitly cites the companion paper for the comprehensive analysis: [Raiola et al., 2025 Quantitative computerized analysis demonstrates strongly compartmentalized tissue deformation patterns underlying mammalian heart tube formation. eLife, under review].

*Comment 8: Also, the Introduction has little number of external references that would be desirable (Blanchard, Bellaiche, Heisenberg, Pastor-Escuredo, etc)*

We thank the reviewer for this suggestion. We have revised the Introduction to include references to the foundational work of Blanchard, Bellaïche, Heisenberg, and Pastor-Escuredo, better positioning our study within the broader context of tissue mechanics and quantitative morphogenesis. We added these citations in the Introduction section line 51-61.

Minor

*“exhibits significant in” this phrase looks incomplete*

Thank you for spotting this typo. We have corrected the sentence.

Responses to Reviewer #2

We thank Reviewer #2 for their positive feedback and for helping us improve the clarity of our methods section.

*Comment 1: On page 31, in the image preprocessing section, the authors mention resizing their 3D images to achieve isotropic resolution, but they don't specify what that resolution actually is.*

We thank the reviewer for the valuable feedback, which has helped us improve the clarity of our methods section. We have now clarified in the Materials and Methods, Image Preprocessing section (lines 794–799) that the images were resampled to achieve an isotropic resolution determined by the native in-plane resolution (x,y) of each specific image, as reported in Table S1 (VoxelRes column). We now explicitly state that the resulting isotropic voxel size is not a single fixed value across all datasets but corresponds to the original in-plane resolution (e.g., 0.593 × 0.593 × 0.593 µm or 0.64 × 0.64 × 0.64 µm, depending on the acquisition).

*Comment 2: This could be linked to the optimization strategy section, where they aim to reduce the resolution of their 3D images to save computational time. One can assume that this downscaling occurs after achieving isotropic resolution, and that all axes are rescaled uniformly — but this is not clearly stated.*

We thank the reviewer for this constructive suggestion. The reviewer’s assumption is entirely correct. We have now clarified in the main text (lines 150–159) that the downscaling step for the optimization strategy is performed after the images have been made isotropic. We explicitly state that the MIRT algorithm and the subsequent optimization method are applied to the fully preprocessed, isotropic images. We have updated the manuscript to make this procedural order unambiguous.

*Comment 3: Additionally, Figure 2F doesn't help clarify this process. In fact, it seems to suggest that rescaling increases computational time, which contradicts the authors’ stated goal. In that same section, they mention both reducing image resolution by 10–25%, and that computational time increases with higher resolutions (10–25%) — which creates some confusion. It seems like this part needs to be rephrased or clarified, as the current wording is somewhat contradictory.*

We thank the reviewer for this important observation. We fully agree, the explanation of the optimization strategy was somewhat confusing.

We have substantially revised the manuscript to clarify our methodology and goal. Our objective was not simply to downscale, but rather to identify the optimal trade-off between computational cost and registration accuracy. We have updated both the 'Optimization Strategy' section (lines 250-275) and the corresponding technical details in the Materials and Methods section (lines 836-847).

The analysis presented in Figure 2F involved testing four levels of progressively increasing image resolution (from a 10% to a 25% setting). The confusion arose because the figure correctly shows that as we use more detailed images, the computational time naturally increases. Our aim was to pinpoint the highest resolution we could use that provided excellent accuracy without an excessive computational burden.

We are confident that these revisions, combined with an updated legend for Figure 2F, now make our optimization strategy and the interpretation of the results entirely unambiguous.

Have the authors made all data and (if applicable) computational code underlying the findings in their manuscript fully available?

The PLOS Data policy requires authors to make all data and code underlying the findings described in their manuscript fully available without restriction, with rare exception (please refer to the Data Availability Statement in the manuscript PDF file). The data and code should be provided as part of the manuscript or its supporting information, or deposited to a public repository. For example, in addition to summary statistics, the data points behind means, medians and variance measures should be available. If there are restrictions on publicly sharing data or code —e.g. participant privacy or use of data from a third party—those must be specified.

*Reviewer #1: No: I think that authors should make more data available on the statistical model and descriptors. It is not so evident to reproduce this from the code just with raw data.*

We thank the reviewer for the comment. To ensure reproducibility, we have uploaded all the necessary input/output data in the Code_Data.zip folder on Mendeley. Since the pipeline requires some manual steps, it cannot be run fully in one single execution; however, each section can be reproduced using the provided data. We also updated the GitHub repository with relative paths to this folder to make the process more manageable.

*Reviewer #2: Yes*
